# Self-efficacy and psychosocial considerations of obesity risk reduction behaviors in young adult white Americans

**DOI:** 10.1371/journal.pone.0235219

**Published:** 2020-06-24

**Authors:** Doreen Liou, Lauren Kulik

**Affiliations:** Department of Nutrition and Food Studies, Montclair State University, Montclair, New Jersey, United States of America; San Raffaele Roma Open University, ITALY

## Abstract

The obesity epidemic is a pervasive health issue affecting all population groups in developed countries. The purpose of this research was to ascertain obesity risk reduction behaviors and their psychosocial determinants in young adult Americans residing in New Jersey state. A cross-sectional survey design was implemented in which a convenience sample of 174 participants (18 to 40 years) completed a validated online self-administered questionnaire. Nineteen obesity risk reduction behaviors, self-efficacy and psychosocial constructs derived from the Theory of Planned Behavior were measured. Statistical analyses were conducted using frequency distributions, t-tests and regression analysis. Regression analysis indicated that 37.5% of the variance in obesity risk reduction behavior was accounted by self-efficacy alone. T-test comparisons indicated greater frequency of adoption of 17 health behaviors among individuals categorized in the ‘high self-efficacy’ group (p<0.05). These behaviors included limiting portion sizes of food, eating fruits and vegetables, engaging in physical activity, and monitoring stress and body weight. Nutrition professionals working with young adult Americans need to assess their self-efficacy to engage in obesity risk reduction behaviors. In fostering confidence in adopting these behaviors, executing skill building nutrition interventions is critical for obesity prevention.

## Introduction

The obesity epidemic is a pervasive global health problem affecting all population groups [1]. Obesity rates among American adults over 20 years of age is 38.6% for the general population [2]. Within the white American population, 36.9% of men and 38.8% of women over the age of 20 are obese [3]. More alarmingly is the prevalence rate of obesity among white American youth [4]. Body mass index (BMI, kg/m^2^) is used to classify individuals into categories of underweight, overweight, and obese. A body mass index of 25 to 29.9 signifies overweight and 30 and above designates various levels of obesity [5].

In the United States, traditional diets rich in complex carbohydrates and fiber have often been replaced with diets high in fat and refined carbohydrates. Globalization and acculturation to the Western lifestyle have been linked to fewer home cooked meals, increased consumption of fast foods and snacking [6]. Changes in patterns of physical activity that are related to obesity risk include increased motorized transport and sedentary recreation and fewer allowances for recreational physical activity [7]. Obesity risk reduction behaviors include consumption of plant-based foods, moderation of portion sizes and limiting intakes of high-fat and sugary foods and beverages [8–10].

Health behavior theories draw upon psychology and sociology to form a foundation for understanding health problems such as the prevention of obesity, developing appropriate interventions, and evaluating their effectiveness [11,12]. The Theory of Planned Behavior (TPB) explores the relationship between behavior and beliefs, attitudes, and intentions [13]. According to Ajzen, behavioral intention is the chief determinant of behavior. Behavioral intention is influenced by three components: (a) attitude toward performing a behavior (behavioral beliefs weighted by evaluation of outcomes), (b) subjective norms (whether important others approve or disapprove of the individual performing the behavior weighted by motivation to comply), and (c) perceived behavioral control. Perceived behavioral control reflects the belief that an individual has and can exercise control over enacting a behavior. Support for the Theory of Planned Behavior’s ability to predict dieting and healthy eating in adult populations has been well documented in the literature [14,15]. Obesity risk reduction behaviors such as increasing fruit and vegetable consumption were among the theory’s best-predicted behaviors along with predicting lower fat consumption [16].

The concept of self-efficacy from the Social Cognitive Theory is foundational in behavioral interventions [17]. Self-efficacy, or the belief in one’s ability to perform a task in a situation, is a behavior-specific construct, which plays a major role in the behavior change process. Perceptions of one’s ability to overcome difficulties in a specific task often predicts future attempts to engage in behavioral challenges.

The purpose of our study was to investigate obesity risk reduction behaviors and its psychosocial determinants in a convenience sample of adult white Americans residing in New Jersey (18 to 40 years of age) with varying weight categories. The psychosocial constructs investigated were derived from the Theory of Planned Behavior and self-efficacy. The researchers hypothesize that behavioral intention and self-efficacy would emerge as significant contributors of obesity risk reduction behaviors. The investigators also sought to determine whether participants’ level of self-efficacy differ in the behaviors measured.

## Materials and methods

This cross-sectional survey investigated 186 free-living white Americans between the ages of 18 and 40 years living in New Jersey. Data were collected from June 2018 to May 2019 from various counties in northern and central New Jersey. Participants were solicited from various associations including educational institutions, churches, and cultural centers providing a diverse range of socioeconomic status and educational backgrounds. In order to ensure statistically significant outcomes per number of variables for the use of the multiple regression technique, a minimum sample size of 150 was sought. A raffle drawing for $25 and $50 gift cards was offered as an incentive for participation. A total of 186 participants completed an online survey. Twelve surveys with incomplete or missing data were discarded, resulting in a final sample size of 174. Montclair State University’s Institutional Review Board approved of this study.

### Measures

The survey instrument used in this investigation was systematically developed by Liou & Bauer [10] from a qualitative pilot study and review of literature. The questionnaire contained 85 questions measuring obesity risk reduction behaviors, psychosocial components, and demographic factors. Demographic information was solicited such as participants’ birthplace, gender, age, education levels, marital and working status, and income. Respondents provided self-reported weight and height, physical activity levels (‘sedentary’ to ‘heavy activity’) and perceived stress levels (‘very stressed’ to ‘very calm’). The respondents took an average of 15 minutes to complete the online survey. The participants’ BMIs were grouped into categories based on calculations of weight and height. The categories included underweight (BMI < 18.5), normal (18.5 ≤ BMI < 25), overweight (25 ≤ BMI <30), and obese (BMI ≥ 30).

#### Obesity risk reduction behaviors

Nineteen questions measured five domains of obesity risk reduction behaviors over the previous month using a scale of 1 to 4 (never/rarely to always/usually). The five behavioral domains in this category included the following contexts: food (9 items), eating behavior (4 items), physical activity (2 items), psychological context (2 items), and knowledge awareness (2 items). The ‘food’ context encompassed eating fruits, vegetables, whole grains, and healthful snacks and pre-packaged foods (e.g.: frozen foods). This context also reflected the selection of steamed foods and limiting high calorie beverages and amounts of fat in cooking. The ‘eating behavior’ context entailed eating home-cooked meals, using smaller portion sizes, and following healthful food patterns. The ‘physical activity’ context assessed exercising regularly and engaging in physically active leisure activity. The ‘psychological’ context measured taking time to relax and improve emotional well-being and decrease stress. Lastly, the ‘knowledge awareness’ context reflected monitoring body weight and learning about obesity prevention. These five domains reflected findings from the literature on obesity prevention and items were amended for their applicability with adult Americans (2,8,9). Questions by domain are outlined in Table 1.

**Table 1:** Examples of questionnaire items.

***Obesity Risk Reduction Behaviors***	
In the past month, how often did you engage in the following behaviors:	
*Food context*	• Ate steamed foods instead of fried foods?
	• Used small amounts of oils or fat when preparing or cooking foods?
	• Ate at least 3 servings of vegetables per day?
	(1 serving = ½ cup cooked, 1 cup fresh leafy vegetables)
	• Ate at least 2 servings of fruits each day?
	(1 serving = 1 medium fruit)
	• Ate at least 3, 1-ounce servings of whole grains per day?
	• Made healthier choices at fast food restaurants?
	• Ate healthful snacks (e.g.: fruit, nuts, etc.)?
	• Ate healthful pre-packaged foods (e.g.: frozen foods)
	• Limited intake of high calorie beverages (e.g.: soft drinks, juice, alcoholic drinks)?
*Eating behavior context*	• Ate home-cooked meals over restaurant-prepared foods?
	• Ate smaller portion sizes of foods than usual?
	• Followed healthful food patterns (e.g.: eating more fruits and vegetables, less red meat)?
	• Used portion size control methods to help decide how much to eat?
*Physical activity context*	• Exercised at least 30 minutes, 3 to 5 days per week (e.g. walking, biking)?
	• Engaged in at least 1 physically active leisure activity?
*Psychological context*	• Took time to relax and improve my emotional well-being? (e.g.: social involvement, positive thinking)
	• Took time to relax to decrease the amount of stress I feel?
*Knowledge awareness context*	• Monitored my body weight?
	• Learned about obesity risk and prevention (e.g.: attending seminars, reading health articles, watching health programs on TV)?
**Psychosocial Constructs**	**Questionnaire Statements**
***Theory of Planned Behavior and Self-Efficacy***	
*Behavioral intention*	During the upcoming week, I plan to eat a least 5 servings of fruits and vegetables each day.
*Attitude*	Selecting a lot of fruits and vegetables to eat is….
	(Favorable vs. Unfavorable)
*Normative beliefs*	In general, how much influence do your friends have on your food choices?
*Motivation to comply*	If my friends gave advice on dietary matters, I (would—would not) follow it.
*Perceived behavioral control*	I am in total control of my weight.
	As long as I want to, I can prevent myself from gaining excessive weight.
*Self-Efficacy*	How confident do you feel in your ability to eat a lot of fruits and vegetables?
	How confident are you in consuming small portion sizes of food?

#### Psychosocial variables: Theory of Planned Behavior and self-efficacy

Eleven items were used to measure intention to practice obesity risk reduction behaviors. For example, the item, “During the next week, I plan to choose small portion sizes of food” was rated on a 7-point scale (‘extremely unlikely’ to ‘extremely likely’). Intention was defined as the summated score of the 11 items. Twelve items were selected to measure direct attitude towards various behaviors such as “Eating home-cooked meals instead of restaurant-prepared foods is ‘favorable’ to ‘unfavorable’ based on a 7-point Likert-type scale.

A total of five categorized groups were listed by American adults during the qualitative study as having a social influence over their health-related practices. They include spouse/partner, friends, parents, children, and physician. Items for normative beliefs were rated on a 7-point scale (‘not at all’ to ‘very much’). Items for motivation to comply were anchored by ‘I would’ to ‘I would not follow it’ on a 7-point Likert-type scale. There was also an inclusion of a “not applicable” category for participants to indicate if these significant others did not exist.

A direct measurement of perceived behavioral control was measured using a total of 2 items. These included questionnaire statements such as “I am in total control of my weight” and “As long as I want to, I can prevent myself from gaining excessive weight.” These items were rated on 5-point scales (‘strongly agree’ to ‘strongly disagree’). Perceived control over performing obesity risk reduction behaviors was defined as the summated score of the 2 items.

Participants indicated their perceived confidence to engage in various obesity risk reduction behaviors (self-efficacy). They included 9 items measuring confidence in consuming fruits/vegetables and healthful snacks, incorporating small portion sizes of food, limiting intake of high calorie beverages, engaging in regular physical activity, monitoring body weight, and engaging in relaxation efforts to reduce stress levels. Response options to the self-efficacy items were rated on 5-point scales anchored by ‘extremely confident’ to ‘not at all confident.’ Participants were dichotomized into two groups based on their mean scores of the 9 items. Individuals with a mean score of 3.5 or greater (range 1 to 5) were designated as ‘high self-efficacy.’ Conversely, participants with mean scores of less than 3.5 were deemed as ‘low self-efficacy.’

#### Questionnaire validity and reliability

A pilot study with 30 American adults from the New York metropolitan area provided clarity and meaning of the questionnaire items. Young professionals and university students (18 to 40 years of age) were queried to ascertain face validity by consistency of their responses with the researchers’ intended meaning of the survey questions. An expert panel of five nutrition behavioral science researchers from various state universities reviewed the contents of the instrument for accurate reflection of constructs. An exploratory factor analysis of principle variables established construct validity. The entire scale produced 9 distinct factors accounting for 62.3% of the variance in responses. After additional factor analysis for each subscale, 6 items had a factor loading of less than 0.40 and were deleted from the scale [18].

The subscale of obesity risk reduction behavior yielded 5 distinct factors accounting for 60.3% of the variance in responses. These distinct factors corresponded conceptually to the 5 contexts of obesity risk reduction behaviors: food, eating behavior, physical activity, psychological context, and knowledge/awareness. Reliability was measured using Cronbach’s alpha internal consistency assessment. The Cronbach’s alpha coefficients of the behavioral variables (0.8) reflected good psychometric properties. Further details of the instrument’s validity and reliability can be found in previously published studies [10,19].

#### Data analysis

All survey data were coded and entered for computer analysis using Statistical Package for Social Sciences, version 25.0. Frequency distributions highlighted the demographic data, ranges of behavior, and psychosocial factors. Pearson’s product-moment correlations were used to examine the significance of the associations (p < .05) between a combined index of obesity risk reduction behaviors and the psychosocial factors. To test our hypothesis, stepwise multiple regression analyses were conducted to determine the subset of psychosocial variables that best predicted the behavioral index. T-tests were performed on demographic factors (e.g.: age, gender, BMI) and self-efficacy groups to determine mean differences in the behaviors measured.

## Results

Approximately 300 survey fliers were distributed to individuals in New Jersey, 174 questionnaires were completed and returned, resulting in a 58% response rate. As shown in Table 2, the mean age of the study participants was 26.4 years (SD = 7.0) with 70.4% females, and 70.5% designated as ‘never married.’ Forty-one percent of respondents completed some college, 43% were college graduates and 16% achieved post-graduate degrees. The average BMI of all participants was 24.7 ± 4.8, with a BMI range of 16.0 to 42.2. Based on BMI categories, 4.3% of the participants were underweight, 46.8% normal weight, 21% overweight and 10.2% were designated as obese. Self-reported stress levels indicated that 22.4% of participants considered themselves very stressed, 51.2% indicated moderately stressed, 18.7% neutral, and approximately 7.7% were moderately to very calm.

**Table 2:** Demographic data of entire sample.

Category	Sample (n = 174)	
Gender, %	Male	29.6
	Female	70.4
Age, years	Mean Age	26.4 ± 7.0
	Range	18 to 40
Body Mass Index, %	Underweight	4.3
	Normal weight	46.8
	Overweight	21.0
	Obese	10.2
	Unreported data	17.7
	Mean BMI	24.7 ± 4.8
Highest Education, %	Elementary school or less	0
	Some high school	0
	High school graduate	0
	Some college	41.0
	College graduate	43.0
	Post graduate	16.0
Stress Level, %	Very Stressed	22.4
	Moderately Stressed	51.2
	Neutral	18.7
	Moderately to Very Calm	7.7
Marital Status, %	Married	18.3
	Widowed	3.0
	Divorced	4.9
	Never Married	70.5
	Domestic Partner	3.3
Physical activity level, %	Sedentary	15.6
	Light activity	37.6
	Moderate activity	36.6
	Heavy activity	10.2

### Obesity risk reduction behaviors

The mean values of the 19 obesity risk reduction behaviors are presented in Table 3. For the entire sample, the mean was $\bar{x}$ = 2.8 ± 0.5 (range of 1 to 4). As a whole, the participants had the highest frequency of engagement in eating home-cooked meals instead of restaurant-prepared foods, eating healthful snacks, limiting intake of high calorie beverages, and following healthful food patterns (i.e.: less red meat & more fruits/vegetables).

**Table 3:** Obesity prevention behaviors for entire sample with t-test comparisons between high vs. low self-efficacy groups.

Category (Score Range = 1 to 4)	Entire SampleMean (SD) (n = 174)	High Self-Efficacy Group Mean (SD) (n = 118)	Low Self-Efficacy Group Mean (SD) (n = 56)	Sig. (2-tailed)
**Psychological**				
Took time to decrease the amount of stress I feel	2.24	2.66	2.24	**p = 0.007
	(0.87)	(0.96)	(0.87)	
Took time to relax and improve my emotional well-being	2.17	2.84	2.17	***p<0.001
	(0.88)	(0.90)	(0.88)	
**Physical Activity Context**				**p = 0.005
Engaged in at least 1 physically active leisure activity	2.63	3.11	2.63	
	(1.00)	(1.01)	(1.00)	
Exercised at least 30 minutes, on 3–5 days/week	2.26	2.95	2.26	***p<0.001
	(0.97)	(1.15)	(0.97)	
**Eating Context**				
Ate home-cooked meals instead of restaurant-prepared meals	3.22	3.53	3.22	*p = 0.044
	(1.00)	(0.73)	(1.00)	
Limited my portion sizes of foods	2.07	2.80	2.07	***p<0.001
	(0.75)	(0.83)	(0.75)	
Used portion size control methods to help decide how much to eat	1.92	2.63	1.92	***p<0.001
	(0.82)	(1.09)	(0.82)	
Followed healthful food patterns	2.52	3.42	2.52	***p<0.001
	(0.88)	(0.82)	(0.88)	
**Food Context**				
Ate steamed foods instead of fried foods	2.44	3.27	2.44	***p<0.001
	(0.90)	(0.85)	(0.90)	
Used small amounts of oils or fat when preparing or cooking foods	2.28	3.08	2.28	***p<0.001
	(1.00)	(0.95)	(1.00)	
Ate at least 3, 1-oz servings of whole grains per day	2.65	2.93	2.65	p = 0.054
	(0.41)	(0.86)	(0.91)	
Ate at least 2 servings of fruit each day	2.44	3.22	2.44	***p<0.001
	(1.02)	(0.93)	(1.02)	
Ate at least 3 servings of vegetables per day	2.44	3.18	2.44	***p<0.001
	(0.96)	(0.92)	(0.96)	
Made healthier choices at fast food restaurants	1.93	2.41	1.93	**p = 0.008
	(0.99)	(1.27)	(0.99)	
Ate healthful snacks	2.87	3.47	2.87	***p<0.001
	(0.87)	(0.72)	(0.87)	
Ate healthful pre-packaged foods	2.20	2.73	2.20	**p = 0.001
	(0.86)	(1.02)	(0.86)	
Limited intake of high-calorie beverages	2.78	3.45	2.78	***p<0.001
	(1.02)	(0.91)	(1.02)	
**Knowledge Awareness Context**				
Monitored my weight	2.09	2.63	2.09	**p = 0.003
	(1.03)	(1.11)	(1.03)	
Learned about obesity risk and prevention	2.24	2.50	2.24	p = 0.179
	(1.10)	(1.19)	(1.10)	

Bold p-values indicate Bonferoni adjusted threshold for statistical significance.

### Regression analysis and correlations

Multiple regression analyses were conducted to determine the subset of TPB variables and self-efficacy that best predicted behavioral intention and behavior. A stepwise procedure was used in each regression analysis with a required probability of entrance into the regression at p<0.05. The five domains of behavior were combined into a single index by taking the mean value of all 19 items to provide a composite measure of obesity risk reduction behaviors. Self-efficacy was not dichotomized in these regression analyses. Self-efficacy (β = 0.61) emerged as the single most prominent predictor, accounting for 37.5% of the variance of this behavioral index. A variance of 19.1% was reported in predicting behavioral intention in which self-efficacy was also the only significant contributor (Table 4).

**Table 4:** Regression analysis of theory of planned behavior and self-efficacy variables in predicting obesity risk reduction behaviors and behavioral intention.

The Predicted	Significant Predictors	R^2^ (%)	β	b	SE of B	p-value
**Obesity Risk Reduction Behaviors**	Self-efficacy	37.5	0.613	0.449	0.102	<0.001
**Behavioral Intention**	Self-efficacy	19.1	0.437	0.695	0.249	0.009

**Fig 1:**
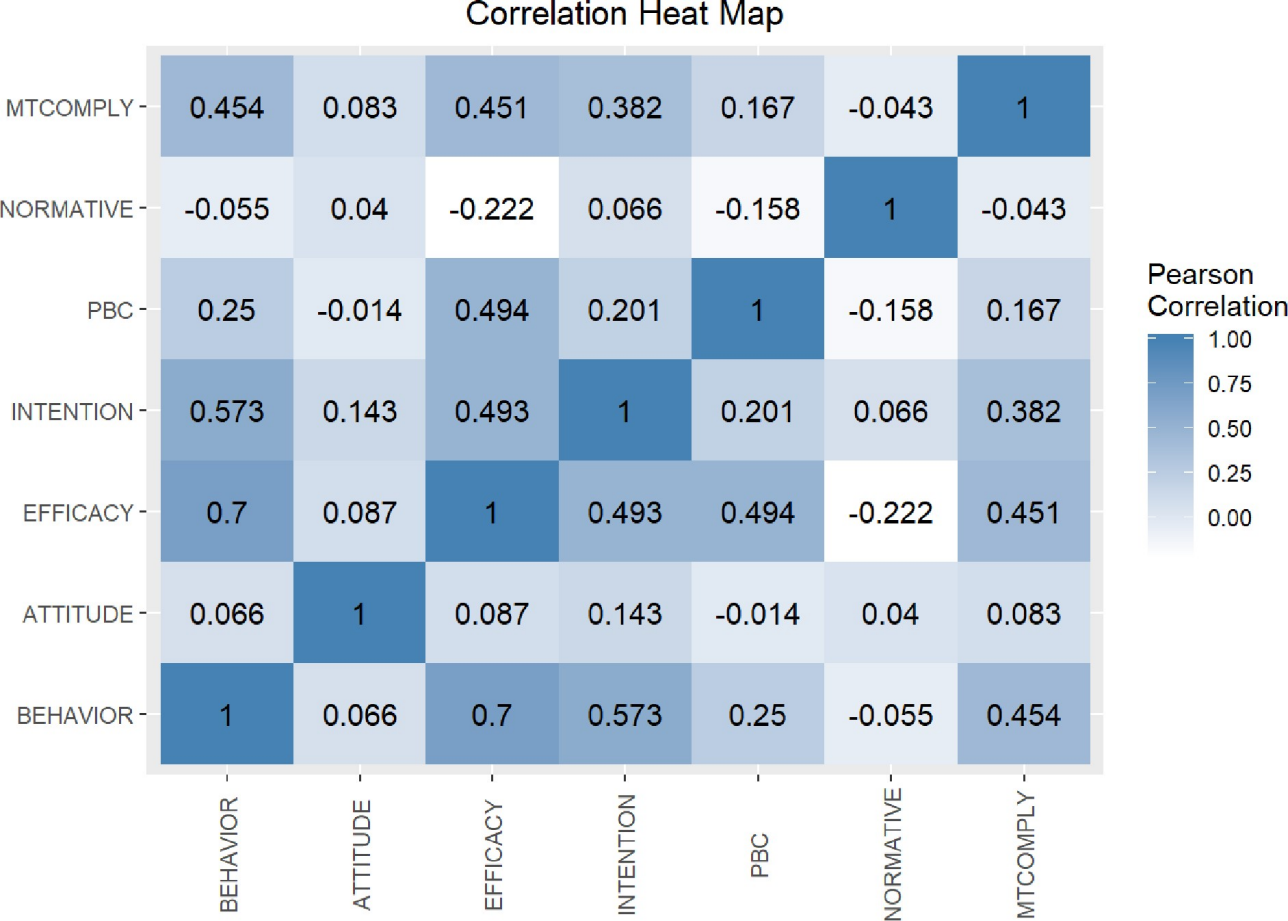
Heatmap of Pearson’s correlations of behavior and psychosocial variables.

Pearson’s correlations were computed between behavior and the psychosocial variables. Self-efficacy was strongly correlated with behavior (r = 0.7, p<0.001). Behavioral intention (r = 0.6, p<0.001), perceived behavioral control (r = 0.3, p<0.01), and motivation to comply (r = 0.5, p<0.01) were also significantly related to behavior (Fig 1). Applying a Bonferoni-based multiple testing correction, an adjusted threshold of 0.0024 would result in the p-values, thereby showing self-efficacy and behavioral intention as statistically significant correlates of behavior.

### T-tests

Participants were divided into two groups according to mean scores of overall self-efficacy. Respondents with high levels of self-efficacy (mean score ≥ 3.5, range 1 to 5) were more likely to practice obesity risk reduction behaviors as compared with individuals with low levels of self-efficacy (mean score < 3.5) Table 3. Out of a total of 19 behaviors, t-tests indicated that 17 behaviors were statistically significant with mean values greater in the high self-efficacy group. These behaviors included eating home-cooked meals instead of restaurant-prepared foods (p<0.05), limiting portion sizes of foods (p<0.001), following healthful food patterns (p<0.001), using portion size control methods to decide how much to eat (p<0.001), eating steamed foods instead of fried foods (p<0.001), limiting amounts of oils or fats (p<0.001), eating at least 3 servings of vegetables per day (p<0.001), eating at least 2 servings of fruits per day (p<0.001), making healthier food choices at fast food restaurants (p<0.01), eating healthful snacks (p<0.001), eating healthful pre-packaged foods (p<0.01), limiting high-calorie beverages (p<0.001), monitoring body weight (p<0.01), exercising at least 30 minutes, 3 to 5 days per week (p<0.001), engaging in at least 1 physically active leisure activity (p<0.01), taking time to relax to improve emotional well-being (p<0.001), and taking time to relax to decrease stress (p<0.01). Applying a Bonferoni-based multiple testing correction, an adjusted threshold of 0.0026 would result in the p-value. Therefore, 12 out of the 19 obesity risk reduction behaviors would remain statistically significant.

T-tests were also conducted with sub-groups of individuals based on age, gender, and BMI categories. Younger participants (ages 18 to 29) ate more healthful pre-packaged foods (Table 3. For the entire sample, the mean was $\bar{x}$ = 2.68 ± 0.97) than older participants (ages 30 to 40) (Table 3. For the entire sample, the mean was $\bar{x}$ = 2.31 ± 0.98, p<0.05). Younger respondents also took more time to relax to decrease stress (Table 3. For the entire sample, the mean was $\bar{x}$ = 2.58 ± 0.93) than their counterparts (Table 3. For the entire sample, the mean was $\bar{x}$ = 2.19 ± 0.94, p<0.05). Statistically significant differences were seen between male versus female participants in their practice of obesity risk reduction behaviors. Females reported increased frequency of eating steamed foods instead of fried foods (p<0.05), using limited amounts of oils or fats (p<0.01), and eating healthful pre-packaged foods (p<0.05). As participants were dichotomized between normal BMI versus overweight and/or obese, only one behavior differentiated the groups. Individuals with normal BMI reported higher frequency of limiting amounts of oils or fat (Table 3. For the entire sample, the mean was $\bar{x}$ = 3.00 ± 0.98) as compared with their counterparts (Table 3. For the entire sample, the mean was $\bar{x}$ = 2.61 ± 1.03, p<0.05).

## Discussion and conclusions

The focus of our study was to analyze the role of psychosocial determinants stemming from the Theory of Planned Behavior and self-efficacy and their relationships with obesity prevention behaviors in white Americans. Our results showed that self-efficacy emerged as the most prominent contributor in regression analyses to predict behavior and behavioral intention. Our research study has practical implications due to the identification of psychosocial constructs, namely self-efficacy, as a moderating factor that needs to be considered when designing nutrition interventions. The concept of self-efficacy is foundational in behavior change interventions. Individuals begin pursuing goals with varying levels of self-efficacy, and higher self-efficacy is generally associated with greater effort and commitment to adopting healthy behaviors [20]. Self-efficacy is enhanced when individuals successfully achieve their goals, propelling the likelihood that behavior change will be maintained [21]. Cha et al. [22] also found positive correlations between healthy eating behavior and self-efficacy in young adults aged 18 to 29. Individuals with higher self-efficacy were more likely to read food labels, thus positively impacting their dietary quality. This research has important applications since the growing trend of obesity among young adults is often characterized by excessive fast food and sugar-sweetened beverage consumption [23].

Our study found stark contrasts in the performance of health behaviors according to individuals’ perceptions of self-efficacy. Individuals with strong levels of behavioral confidence had higher frequency of consumption of plant-based foods with greater engagement in physical activity than the ‘low self-efficacy’ group. The ‘high self-efficacy’ group also moderated their stress levels and limited portion sizes of food more than their counterparts.

The Theory of Planned Behavior has been shown to be a strong predictor of a wide range of health behaviors including physical activity [24,25]. Based on TPB meta-analyses, intention accounted for approximately 25% of variance of a variety of health behaviors [15]. In predicting intention as a dependent variable, attitudes, subjective norm, and perceived behavioral control accounted for approximately 39% to 44% of the variance [12,15]. Studies incorporating TPB variables in the prediction of food-related behaviors have found attitudes to predominantly predict behavioral intention [26–28]. Blanchard et al. [29] reported intention for fruit and vegetable consumption was significantly predicted by attitudes and perceived behavioral control in white Americans.

Liou & Bauer [19] found that self-efficacy, intention, and attitudes accounted for 38% to 47% of the variance of obesity risk reduction behaviors among Chinese Americans living in both Los Angeles county (California) and in the New York metropolitan areas, respectively. Individuals with a favorable disposition or attitude towards healthy eating behaviors were likely to engage in these dietary practices. Other researchers have demonstrated that individuals with a positive affective response (e.g.: attitude toward exercise) not only increased motivation to exercise, but also sustained motivation over time [25,30]. Interestingly, in our study with white Americans, attitude did not emerge as a significant contributor of behavior. Self-efficacy alone accounted for 37.5% of the variance of obesity risk reduction behaviors.

Gender differences in the practice of obesity risk reduction behaviors were also differentiated in our study. Females were seen to adopt more healthy food preparations such as steaming and using less oils/fats than their male counterparts. This was consistent with findings by other researchers [31,32] indicating a stronger motivation to engage with health-related information and behavioral practices than males.

Several limitations are acknowledged that might temper interpretation of our findings. Firstly, our sample represented a convenience sample of young, relatively healthy white American individuals. The use of a convenience sample as opposed to a random sample limits generalizability, warranting replications. Our sample is not demographically representative of the American white population in terms of education level and sex. Because the sample was a convenience one, generalization to other more diverse populations is difficult. Secondly, the self-report nature of obesity risk reduction behaviors, height, and weight is also a limitation. The underreporting of actual weight and height may account for lower prevalence of obesity found in our study. Although the validity of self-reports appeared to be satisfactory, supplementation by more objective measures of behavior and anthropometric measurements is desirable. Furthermore, longitudinal studies are needed that include more measured points in time. Lastly, an increase in family-wise error rate across the reported statistical analyses would moderate t-test results for age, gender, and BMI sub-groups. The researchers of this study encourage replication in future investigations.

Despite these caveats, our study has important implications for dietitians and nutrition educators to plan and execute skill-building interventions critical for American millennials. It is important to raise awareness and confidence to successfully engage in obesity risk reduction behaviors, especially for young adult males and older versus younger individuals. These specific dietary behaviors include limiting portion sizes and selecting healthful food choices at eateries. In addition, nutrition education can foster culinary skills among young adults in creating tasty, home-cooked meals with less fat. Nutrition interventions can empower overweight/obese individuals to choose plant-based options (e.g.: fruits, vegetables, and whole grains) in real-life settings where young adults are easily accessible. The results of this study may aid in the design of more effective interventions, especially with respect to encouraging stable intentions for healthy eating and exercise behaviors and greater behavior maintenance. We need a better understanding of the inter-relationships among Theory of Planned Behavior constructs and self-efficacy in facilitating behavior change. A transdisciplinary approach is warranted with careful consideration of genetic, physiological and environmental factors.

## Supporting information

S1 File (DOCX)
