# Supplementary material for: Self-efficacy and psychosocial considerations of obesity risk reduction behaviors in young adult white Americans
Source: PLoS One. 2020 Jun 24;15(6):e0235219. doi: 10.1371/journal.pone.0235219 (PMC7314022; doi:10.1371/journal.pone.0235219)
Supplement: S1 File — (DOCX) [file pone.0235219.s001.docx]

***SURVEY***

***HEALTH & FOOD BELIEFS***


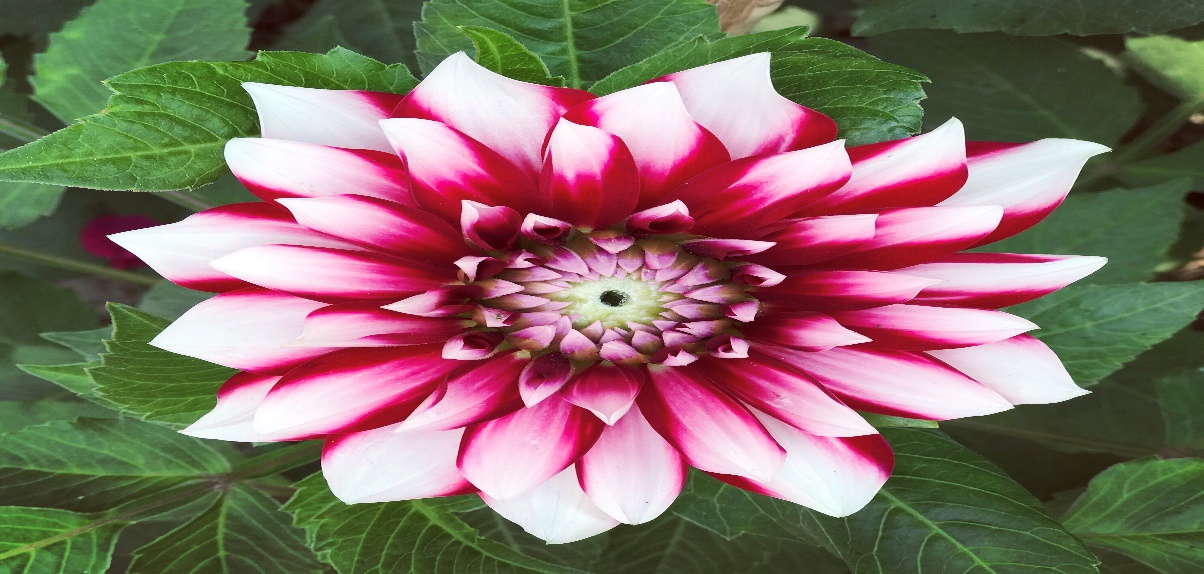


**MONTCLAIR STATE UNIVERSITY**

**Nutrition researchers at Montclair State University are conducting a health and food beliefs survey. Your opinions are very important and can help further the knowledge of health professionals serving the adult American community. Your participation in this study is entirely voluntary and all responses to these questions will be kept confidential.**

**Thank you.**

**Please complete and return this survey to:**

**Doreen Liou, EdD, RDN**

**Montclair State University**

**1 Normal Avenue**

**University Hall 4022**

**Montclair, New Jersey 07043**

**2018/2020**

**A. In the past MONTH, how often did you engage in the following behaviors? Please circle.**

R**arely or Always or Not**

**Never** **Sometimes Often** U**sually Applicable**

| **1** | Ate home-cooked meals instead of restaurant-prepared foods | **1** | **2** | **3** | **4** | **NA** |
| --- | --- | --- | --- | --- | --- | --- |
| **2** | Limited my portion sizes of foods | **1** | **2** | **3** | **4** | **NA** |
| **3** | Ate steamed foods instead of fried foods | **1** | **2** | **3** | **4** | **NA** |
| **4** | Used limited amounts of oils or fat when preparing or cooking foods | **1** | **2** | **3** | **4** | **NA** |
| **5** | Ate at least 3 servings of vegetables per day  (1 serving = ½ cup cooked, 1 cup fresh leafy veg.) | **1** | **2** | **3** | **4** | **NA** |
| **6** | Ate at least 2 servings of fruits each day  (1 serving = 1 medium fruit) | **1** | **2** | **3** | **4** | **NA** |
| **7** | Ate at least 3, 1-ounce servings of whole grains per day | **1** | **2** | **3** | **4** | **NA** |
| **8** | Made healthy choices at fast food restaurants | **1** | **2** | **3** | **4** | **NA** |
| **9** | Ate healthful snacks (e.g.: fruit, nuts, etc.) | **1** | **2** | **3** | **4** | **NA** |
| **10** | Ate healthful pre-packaged foods | **1** | **2** | **3** | **4** | **NA** |
| **11** | Took time to relax and improve my emotional well-being  (e.g.: social involvement, positive thinking) | **1** | **2** | **3** | **4** | **NA** |
| **12** | Took time to relax to decrease the amount of stress I feel | **1** | **2** | **3** | **4** | **NA** |
| **13** | Followed healthful food patterns  (e.g.: eating more fruits & vegetables, less red meat) | **1** | **2** | **3** | **4** | **NA** |
| **14** | Used portion size control methods to help decide how much to eat | **1** | **2** | **3** | **4** | **NA** |
| **15** | Limited intake of high calorie beverages  (e.g.: soft drinks, juice, alcoholic drinks) | **1** | **2** | **3** | **4** | **NA** |
| **16** | Monitored my body weight | **1** | **2** | **3** | **4** | **NA** |
| **17** | Exercised at least 30 minutes, on 3 to 5 days per week  (e.g. walking, biking) | **1** | **2** | **3** | **4** | **NA** |
| **18** | Learned about obesity risk and prevention (e.g.: attending seminars, reading health articles, watching health programs on TV) | **1** | **2** | **3** | **4** | **NA** |
| **19** | Engaged in at least 1 physically active leisure activity | **1** | **2** | **3** | **4** | **NA** |

**B. For each statement, please check the response that best describes your attitude.**

| **1** | Eating home-cooked meals instead of restaurant-prepared foods is…  Favorable __ : __ : __ : __ : __ : __ : __ Unfavorable |
| --- | --- |
| **2** | Choosing small portion sizes of foods is…  Favorable __ : __ : __ : __ : __ : __ : __ Unfavorable |
| **3** | Following traditional healthful Chinese food patterns (e.g.: eating more fruits & vegetables, less red meat) instead of typical Western food habits is…  Favorable __ : __ : __ : __ : __ : __ : __ Unfavorable |
| **4** | Using large amounts of cooking oils or fat in preparing meals is…  Favorable __ : __ : __ : __ : __ : __ : __ Unfavorable |
| **5** | Limiting my intake of high calorie beverages (e.g.: soft drinks, juice, alcoholic drinks) is…  Favorable __ : __ : __ : __ : __ : __ : __ Unfavorable |
| **6** | Engaging in relaxation efforts to reduce stress levels is…  Favorable __ : __ : __ : __ : __ : __ : __ Unfavorable |
| **7** | I consider doing regular physical activity each week as…  Favorable __ : __ : __ : __ : __ : __ : __ Unfavorable |
| **8** | Choosing steamed instead of fried foods is…  Favorable __ : __ : __ : __ : __ : __ : __ Unfavorable |
| **9** | Personal monitoring of my weight is…  Favorable __ : __ : __ : __ : __ : __ : __ Unfavorable |
| **10** | Selecting a lot of fruits and vegetables to eat is…  Favorable __ : __ : __ : __ : __ : __ : __ Unfavorable |
| **11** | Eating my favorite high-calorie foods at a fast food restaurant is …  Favorable __ : __ : __ : __ : __ : __ : __ Unfavorable |
| **12** | Eating high-calorie junk foods is…  Favorable __ : __ : __ : __ : __ : __ : __ Unfavorable |

**C. For each statement, please circle the response that best describes your belief. Please circle.**

**Extremely Very Moderately Slightly Not at all**

**Confident Confident Confident**

| **1** | How confident are you in consuming small portion sizes of foods? | **1** | **2** | **3** | **4** | **5** |
| --- | --- | --- | --- | --- | --- | --- |
| **2** | If you went to a restaurant, how confident do you feel about selecting foods that are not fried? | **1** | **2** | **3** | **4** | **5** |
| **3** | How confident do you feel in your ability to eat a lot of fruits and vegetables? | **1** | **2** | **3** | **4** | **5** |
| **4** | How would you assess your ability to limit intake of high calorie beverages (e.g.: soft drinks, juice, alcoholic drinks)? | **1** | **2** | **3** | **4** | **5** |
| **5** | How confident are you to perform regular physical activity for at least 30 minutes, 3 to 5 days per week? | **1** | **2** | **3** | **4** | **5** |
| **6** | How would you assess your ability to use relaxation efforts to reduce your stress levels? | **1** | **2** | **3** | **4** | **5** |
| **7** | How confident do you feel about monitoring your body weight? | **1** | **2** | **3** | **4** | **5** |
| **8** | How confident are you that you can make healthful choices in a fast food restaurant? | **1** | **2** | **3** | **4** | **5** |
| **9** | How confident do you feel in your ability to eat healthy snacks? | **1** | **2** | **3** | **4** | **5** |

**D. Please state what you believe you actually will do, rather than what you think you should do during the upcoming week. Please check.**

**During the next week, I plan to:**

|  | |
| --- | --- |
| **1** | Choose home-cooked meals more often than restaurant-prepared food…  Extremely unlikely __ : __ : __ : __ : __ : __ : __ Extremely likely |
| **2** | Choose small portion sizes of food…  Extremely unlikely __ : __ : __ : __ : __ : __ : __ Extremely likely |
| **3** | Choose steamed foods over fried ones in the upcoming week…  Extremely unlikely __ : __ : __ : __ : __ : __ : __ Extremely likely |
| **4** | Use small amounts of oils and fat when preparing or cooking foods…  Extremely unlikely __ : __ : __ : __ : __ : __ : __ Extremely likely |
| **5** | Eat at least 5 servings of fruits and vegetables each day…  Extremely unlikely __ : __ : __ : __ : __ : __ : __ Extremely likely |
| **6** | Exercise most days…  Extremely unlikely __ : __ : __ : __ : __ : __ : __ Extremely likely |
| **7** | Learn about obesity risk and prevention…  Extremely unlikely __ : __ : __ : __ : __ : __ : __ Extremely likely |
| **8** | Monitor my body weight…  Extremely unlikely __ : __ : __ : __ : __ : __ : __ Extremely likely |
| **9** | Relax, so I can reduce my overall stress level…  Extremely unlikely __ : __ : __ : __ : __ : __ : __ Extremely likely |
| **10** | Make healthy choices at a fast food restaurant…  Extremely unlikely __ : __ : __ : __ : __ : __ : __ Extremely likely |
| **11** | Limit my high-calorie snack intake…  Extremely unlikely __ : __ : __ : __ : __ : __ : __ Extremely likely |

**E. Please circle the number that best describes your response to each of the following statement.**

| **Strongly Agree** | Agree | **Uncertain** | **Disagree** | **Strongly Disagree** | **Not Applicable** |
| --- | --- | --- | --- | --- | --- |
| **SA** | **A** | **U** | **D** | **SD** | **N/A** |

| **1** | I am in total control of my weight. | **SA** | **A** | **U** | **D** | **SD** | **N/A** |
| --- | --- | --- | --- | --- | --- | --- | --- |
| **2** | As long as I want to, I can prevent myself from gaining excessive weight. | **SA** | **A** | **U** | **D** | **SD** | **N/A** |

**F. Please check your answers.**

| **1** | In general, how much influence does your physician have on your food choices?  Not at all __ : __ : __ : __ : __ : __ : __ Very much ___Not Applicable |
| --- | --- |
| **2** | In general, how much influence does your spouse or partner have on your food choices?  Not at all __ : __ : __ : __ : __ : __ : __ Very much ___Not Applicable |
| **3** | In general, how much influence do your friends have on your food choices?  Not at all __ : __ : __ : __ : __ : __ : __ Very much |
| **4** | In general, how much influence do your children have on your food choices?  Not at all __ : __ : __ : __ : __ : __ : __ Very much ___Not Applicable |
| **5** | My parents believe that I should follow healthful food patterns.  Strongly Agree__ : __ : __ : __ : __Strongly Disagree ___Not Applicable |
| **6** | My parents think that I should not eat a lot of food.  Strongly Agree__ : __ : __ : __ : __Strongly Disagree ___Not Applicable |
| **7** | Generally speaking, how much do you want to do what your parents think you should do in regards to dietary matters?  Not at all __ : __ : __ : __ : __ : __ : __ Very much ___Not Applicable |
| **8** | If my friends told me to eat better…  I would __ : __ : __ : __ : __ : __ : __ I would not |
| **9** | If my doctor told me to eat better…  I would __ : __ : __ : __ : __ : __ : __ I would not |
| **10** | If my parents told me to adopt more healthful food patterns…  I would __ : __ : __ : __ : __ : __ : __ I would not ___Not Applicable |
| **11** | If my friends gave advice on dietary matters…  I would __ : __ : __ : __ : __ : __ : __ I would not follow it |
| **12** | If my children gave advice on dietary matters,  I would __ : __ : __ : __ : __ : __ : __ I would not follow it ___Not Applicable |
| **13** | If my spouse or partner tells me to choose healthy foods,  I would __ : __ : __ : __ : __ : __ : __ I would not ___Not Applicable |

**G.** **We'd like some general information about you. Please fill in or circle your responses.**

1. What is the name of the city and country that you were born in?

__________________________________
(City, State)

_________________________

(Country)

1. What is your gender?

Male 1

Female 2

1. What was your age at your last birthday?

    ________________years
2. What is the highest level of formal education you received?

Elementary school or less 1

Some high school 2

High school graduate 3

Some college 4

College graduate 5

Post graduate degree 6

1. What is your marital status?

Married 1

Widowed 2

Divorced 3

Separated 4

Never married 5

Domestic partner 6

1. Are you currently….(Circle all that apply.)

Employed 1

Approximate number of hours
work per week ___________ HRS

Retired/disabled 2

Homemaker 3

College Student 4

Temporarily unemployed 5

1. What is your average personal income level per year?

Under $20,000 1

$20,000-39,999 2

$40,000-59,999 3

$60,000-79,999 4

$80,000 and above 5

1. Describe the neighborhood in which you live most of the time.

High income 1

Middle-to-high income 2

Middle income 3

Low income 4

1. What is the number of individuals living in your household or residence (including self)?

1 individual 1

2 individuals 2

3 individuals 3

4 or more individuals 4

1. Do you have children?

Yes 1

No 2

1. If you answered “Yes” to question 11, how many children live in your household who are…?

Less than 5 years old _______

5 through 12 years old _______

13 through 17 years old _______

1. nd older _______

12. Who is the main individual who prepares food

in your household?

Self 1

Parent(s) 2

Spouse or significant other 3

Children 4

Friend or roommate 5

Foodservice personnel 6

13. What is your current height and weight?

Height = _______feet ______inches

Weight = _______ pounds

14. Rate your overall stress level.

Very stressed 1

Moderately stressed 2

Neutral 3

Moderately calm 4

Very calm 5

15. Which response best describes how you generally spend your leisure time?

**Sedentary** 1

(At rest most of the day,
 sitting, little walking)

**Light Activity** 2
(Standing, laboratory work)

**Moderate Activity** 3

(Walking, occasional sports,
physical exertion)

**Heavy Activity** 4

(Athletic training,
 labor-intensive work)

16. Approximately how many hours per week do you exercise? ____________hours per week

17. “Eating nutritious foods is not possible because of my limited access to healthy foods.” Was that often, sometimes, or never true for you/your household in the last 3 months?

Often true 1

Sometimes true 2

Never true 3

Don’t know 4

18. How do you rate the quality of your health?

Excellent 1

Good 2

Fair 3

Poor 4

**THANK YOU FOR YOUR PARTICIPATION!**
